# Comparing methods to combine functional loss and mortality in clinical trials for amyotrophic lateral sclerosis

**DOI:** 10.2147/CLEP.S153196

**Published:** 2018-03-19

**Authors:** Ruben PA van Eijk, Marinus JC Eijkemans, Dimitris Rizopoulos, Leonard H van den Berg, Stavros Nikolakopoulos

**Affiliations:** 1 Department of Neurology, University Medical Center Utrecht, Utrecht, the Netherlands; 2 Department of Biostatistics, University Medical Center Utrecht, Utrecht, the Netherlands; 3 Department of Biostatistics, Erasmus University Medical Center, Rotterdam, the Netherlands; 4 Department of Neurology, University Medical Center Utrecht, Utrecht, the Netherlands; 5 Department of Biostatistics, University Medical Center Utrecht, Utrecht, the Netherlands

**Keywords:** joint models, CAFS, clinical trials, amyotrophic lateral sclerosis

## Abstract

**Objective:**

Amyotrophic lateral sclerosis (ALS) clinical trials based on single end points only partially capture the full treatment effect when both function and mortality are affected, and may falsely dismiss efficacious drugs as futile. We aimed to investigate the statistical properties of several strategies for the simultaneous analysis of function and mortality in ALS clinical trials.

**Methods:**

Based on the Pooled Resource Open-Access ALS Clinical Trials (PRO-ACT) database, we simulated longitudinal patterns of functional decline, defined by the revised amyotrophic lateral sclerosis functional rating scale (ALSFRS-R) and conditional survival time. Different treatment scenarios with varying effect sizes were simulated with follow-up ranging from 12 to 18 months. We considered the following analytical strategies: 1) Cox model; 2) linear mixed effects (LME) model; 3) omnibus test based on Cox and LME models; 4) composite time-to-6-point decrease or death; 5) combined assessment of function and survival (CAFS); and 6) test based on joint modeling framework. For each analytical strategy, we calculated the empirical power and sample size.

**Results:**

Both Cox and LME models have increased false-negative rates when treatment exclusively affects either function or survival. The joint model has superior power compared to other strategies. The composite end point increases false-negative rates among all treatment scenarios. To detect a 15% reduction in ALSFRS-R decline and 34% decline in hazard with 80% power after 18 months, the Cox model requires 524 patients, the LME model 794 patients, the omnibus test 526 patients, the composite end point 1,274 patients, the CAFS 576 patients and the joint model 464 patients.

**Conclusion:**

Joint models have superior statistical power to analyze simultaneous effects on survival and function and may circumvent pitfalls encountered by other end points. Optimizing trial end points is essential, as selecting suboptimal outcomes may disguise important treatment clues.

## Introduction

Amyotrophic lateral sclerosis (ALS) is an incurable, rapidly progressive, neurodegenerative disease. As ALS significantly reduces the patient’s life expectancy, evaluating the efficacy of experimental treatments in terms of a benefit to survival is the ultimate goal of any ALS clinical trial.^1^ However, survival time may be influenced by life-extending interventions (such as gastrostomy or tracheostomy) and provides little information about the patient’s functioning and disability during life.^2^–^7^ Moreover, measuring survival time requires lengthy and large clinical trials, which may not be suitable during either early or late phases of drug development, especially when one considers the relatively low incidence of ALS.^2^–^6^,^8^

In order to reduce both the sample size and the duration of ALS clinical trials, functional outcome measures, such as the revised amyotrophic lateral sclerosis functional rating scale (ALSFRS-R), are often used as a primary end point.^9^ The ALS-FRS-R is a clinically relevant, easily obtained, well-validated measurement, which is highly predictive of overall survival.^2^–^4^,^6^ However, results from earlier development programs, with positive Phase II results on functional measures, have until now translated poorly into Phase III survival end points.^2^,^4^ Furthermore, it remains a matter of debate how one should manage missing data from functional scores due to death.^1^,^9^,^10^

Although both the functional and survival end points have their own strengths and weaknesses, treatments for ALS may, nevertheless, affect both survival and function simultaneously. Therefore, any trial based on a single end point might only partially capture the full treatment effect and potentially falsely discard the tested agent as futile.^1^,^10^ Several solutions have been suggested and used in past ALS clinical trials to circumvent this issue, with most notably the combined assessment of function and survival (CAFS).^11^,^12^ The CAFS has been shown by simulation to increase statistical power when there is a treatment effect on both the functional and survival end points.^1^,^10^ However, an important limitation of the CAFS is that it becomes underpowered when there is an exclusive survival benefit without functional gain, a scenario seen, for example, in the riluzole trials, which could potentially lead to false-negative conclusions.^1^,^10^,^13^

Joint models (or shared parameter models) are another well-known method to simultaneously analyze functional decline and mortality.^14^ In contrast to the CAFS, joint models can assess treatment effects on both end points individually and may thus potentially overcome the limitations of the CAFS. However, up to now, joint models have only been used to correct for informative censoring in functional scores in ALS and there is no evidence for their capacity to detect treatment effects.^1^,^14^,^15^ Other methods to combine function and mortality (for instance, time to reach a certain disease state or death)^16^ have never been formally validated, and their statistical properties remain unknown. In this simulation study, therefore, we compared multiple strategies for the simultaneous assessment of functional decline and mortality in ALS clinical trials that aimed to show efficacy of new therapeutic interventions.

## Methods

### Patient data

In this study, the Pooled Resource Open-Access ALS Clinical Trials (PRO-ACT) database (version December 2015, available at https://nctu.partners.org/ProACT) was used to obtain realistic estimates for our simulations.^17^ It contains data from 23 trials performed over the past 20 years, is institutional review board approved and uses solely anonymized data. Subjects consented to participate during the individual trials. Only participants randomized in the placebo arm were used in this study. Participants without ALSFRS-R data were excluded from the analysis. For each individual, we matched demographic, ALSFRS-R and survival data; if no survival data were available, subjects were censored after their last known follow-up visit. To make the PRO-ACT patient population comparable to common Phase III trial populations, we excluded patients with a symptom duration, defined as the time between symptom onset and trial enrollment, longer than 36 months; predicted vital capacity <60% or being older than 80 years.

### Disease model

**Figure 1:**
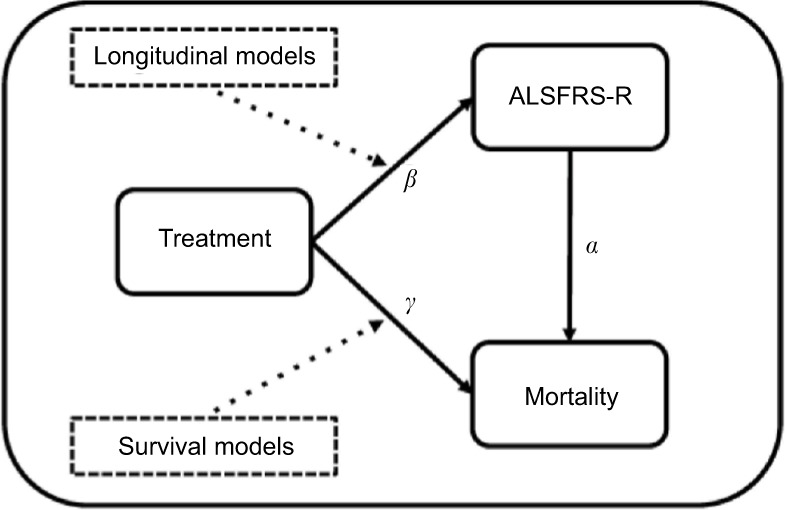
Overview of the relationships between ALSFRS-R, mortality and treatment. **Notes:** In this diagram, treatment can have either a direct effect on mortality by *γ* or an indirect effect on mortality by modifying the ALSFRS-R through *β* and subsequently affecting mortality by *α*. Classically, longitudinal (e.g., linear mixed) and survival (e.g., Cox) models analyze either *γ* or *β*. Joint models incorporate all relationships and simultaneously model *γ*, *β* and *α*. **Abbreviation:** ALSFRS-R, revised amyotrophic lateral sclerosis functional rating scale.

Figure 1 provides a systematic overview of the assumed model underlying the relationship between treatment, functional loss (measured with the ALSFRS-R) and mortality. In this framework, which is a graphical representation of a joint model, treatment can improve overall survival by directly affecting the patient’s hazard (*γ*) or indirectly by reducing ALSFRS-R function loss (*β*) and improving overall survival through the ALSFRS-R (*α*). Classically, survival models evaluate *γ* (expressed as hazard ratio [HR]), while *β* (expressing the reduction in rate of decline) is estimated using linear mixed effects (LME) models. Thus, both methods, if used in isolation, underestimate the full treatment effect by ignoring the effect in either *γ* or *β*. Joint models circumvent this problem by incorporating the ALSFRS-R submodel as a covariate within the survival model and specifically model the relationship between functional loss and mortality through *α*.^14^ Joint models can, therefore, accommodate both the indirect (β * α) and direct (*γ*) treatment effects and form the foundation of our data-generating mechanism.

### Simulation description

A joint model with a Weibull baseline hazard was fitted to the PRO-ACT database; due to selection of ALS trial participants, based on the likelihood they would survive follow-up, death rates accelerated over time and a constant (exponential) death rate could not be assumed (Figure S1). The LME incorporated quadratic random effects for time as this considerably improved the fit to the ALSFRS-R data, *χ*^2^(3) = 1908; the fixed effect of time was modeled linearly as a more complicated model minimally improved the fit, *χ*^2^ (1) = 7. Based on the parameter estimates from the PRO-ACT joint model (Table S1), we generated longitudinal ALSFRS-R measurements and conditional survival data based on a Weibull distribution. A detailed description of the simulation can be found in the “Methods” section of the Supplementary materials. Additionally, we assumed that ~10% of the participants would be lost to follow-up, irrespective of their survival time or functional decline. Based on 50,000 simulations, these settings resulted in a 12- and 18-month survival in the placebo group of 86.5% (95% confidence interval [CI]: 86.1%–86.9%) and 64.2% (95% CI: 63.6%–64.8%), respectively.

### Treatment scenarios

We simulated nine different combinations of treatment effects, based on commonly expected effect sizes in previous ALS clinical trials,^1^,^11^–^13^ where treatment was expected to result in a hazard reduction of 0%, 34% or 50% (HR of 1, 0.66 or 0.5, respectively) and/or an ALSFRS-R slope reduction of 0%, 15% or 30%. All nine scenarios were evaluated when the maximum follow-up duration was either 12 or 18 months, with monthly follow-up visits and a fixed sample size of 150 individuals per treatment arm. For each scenario, we considered the following analytical strategies, which are discussed later: 1) CAFS; 2) Cox model based on the composite end point time-to-6 point decrease on ALSFRS-R or death; 3) omnibus test based on the joint model and 4) omnibus test based on the Cox model and LME model.

### Analytical strategies

All analytical strategies shared the common objective to identify a treatment effect, which resulted in either a beneficial increase in survival or slowing of functional decline. The CAFS ranks each patient according to his or her time to death or functional loss, resulting in one summarized *p*-value for the full treatment effect.^10^ The composite end point time-to-6 point ALSFRS-R decrease or death, an end point used in a recent trial,^16^ is a similar approach and likewise results in one *p*-value for the overall treatment effect. To make a direct comparison with the CAFS and the composite end point, we developed an omnibus test using the joint model framework (Figure 1). The omnibus test was based on two joint models. The first model (JM1) incorporated an LME model without the treatment–time interaction and a Cox model with only an intercept and thus only models *α*. The second joint model (JM2) incorporated an LME model with the treatment–time interaction (*β*) and a Cox model with the treatment indicator as a covariate (*γ*). We used a likelihood ratio test with two degrees of freedom to compare JM1 and JM2 to obtain one *p*-value for the overall treatment effect. A second omnibus test was developed based on the individual LME and Cox models. The treatment effects within both models were tested against a corrected *p*-value to control type 1 error according to the Hommel correction for multiple testing.^18^ Treatment was considered effective when either one of the models was below the threshold. Table 1 provides an overview of the null and alternative hypothesis tested by each of the analytical strategies. Our primary focus was on empirical power, defined as the proportion of simulation samples in which the null hypothesis of no effect was rejected. To improve understanding of our results, we translated empirical power to sample size using the formula provided by Healy and Schoenfeld.^1^ For the CAFS, the composite end point and the joint model test, we used a two-sided alpha of 0.05. In order to complete the comparison, we also provided empirical power for the separate Cox and LME models.

## Results

### PRO-ACT database

**Figure 2:**
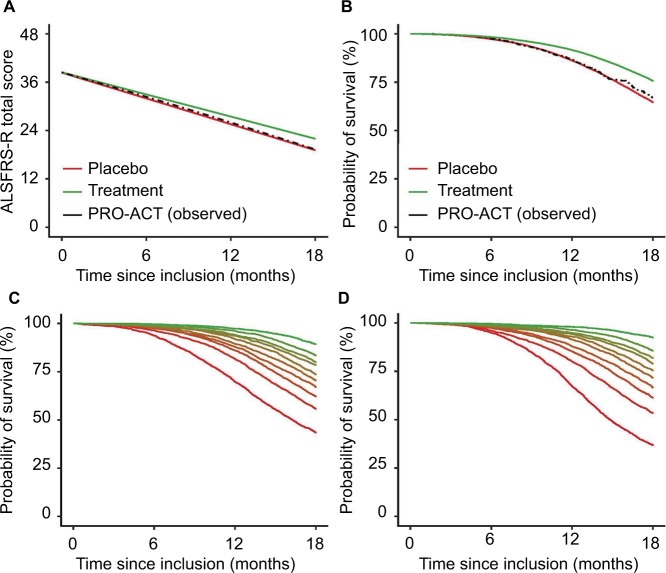
Rates of functional decline and mortality in the PRO-ACT database with a simulated treatment scenario. **Notes:** (**A** and **B**) Observed rates of functional decline and death in the 1,469 selected PRO-ACT patients (Table 2). Our simulation (n = 50,000) shows a good fit with the observed dataset and exhibits a similar pattern over time. As illustration, a hypothetical treatment effect was induced, which reduced the rate of decline by 15% and the hazard by 34%. To illustrate the interaction (*α*) between the ALSFRS-R and survival, we divided subjects into ten equally sized groups according to their ALSFRS-R baseline score (**C**) or observed rate of decline during follow-up (**D**). Green represents the patients with the highest baseline score or the slowest rate of decline. **Abbreviations:** ALSFRS-R, revised amyotrophic lateral sclerosis functional rating scale; PRO-ACT, Pooled Resource Open-Access ALS Clinical Trials.

In total, we selected 1,469 patients with 15,506 ALSFRS-R measurements and a total follow-up time of 1,524 person-years, during which 285 deaths were recorded. Baseline characteristics of the PRO-ACT cohort are given in Table 2 and are comparable with other large trial populations.^11^,^12^ On average, the longitudinal ALSFRS-R rate of decline was 1.06 points (95% CI: 1.01–1.10 points) per month. There is a strong longitudinal relationship between the ALSFRS-R and the risk of death (HR 0.88 [95% CI: 0.87–0.89]; *p* < 0.001), indicating that a one-point increase in ALSFRS-R score reduces the risk of death during follow-up by 12% (Table S1). This relationship underscores the importance of *α* (Figure 1). Figure 2 shows the observed PRO-ACT data and a simulated trial (n = 50,000) with a hypothetical treatment affecting both function and mortality. The simulated placebo group has similar functional and mortality rates as the 1,469 selected PRO-ACT patients (Figure 2A and B). Figure 2C and D visu-alizes the role of *α* and the extent of survival heterogeneity in our simulations: the baseline ALSFRS-R score (Figure 2C) and the rate of ALSFRS-R decline (Figure 2D) both affect the survival probability during follow-up.

### False-positive rates and classical analyses

Empirical power values of each analytical strategy are given in Tables 3 and S2; the first row represents the scenario when there is neither a survival (HR = 1) nor a functional (rate of decline reduction = 0%) benefit to treatment. The false-positive rate (type I error) is approximately 5% for all tests, indicating that all analytical strategies are statistically valid. As expected from Figure 1, both the separate LME and Cox models have increased false-negative rates (type II error = 1 - power) when treatment exclusively affects either functional decline or survival. For instance, power of the LME model remains ~5%, while there is a clear survival benefit in the treatment arm (rows 2 and 3; Table 3). Vice-versa, the Cox model exhibits an increase in the false-negative rate when there is a clear functional benefit (rows 4 and 7; Table 3).

### Comparison of combined analytical strategies

**Figure 3:**
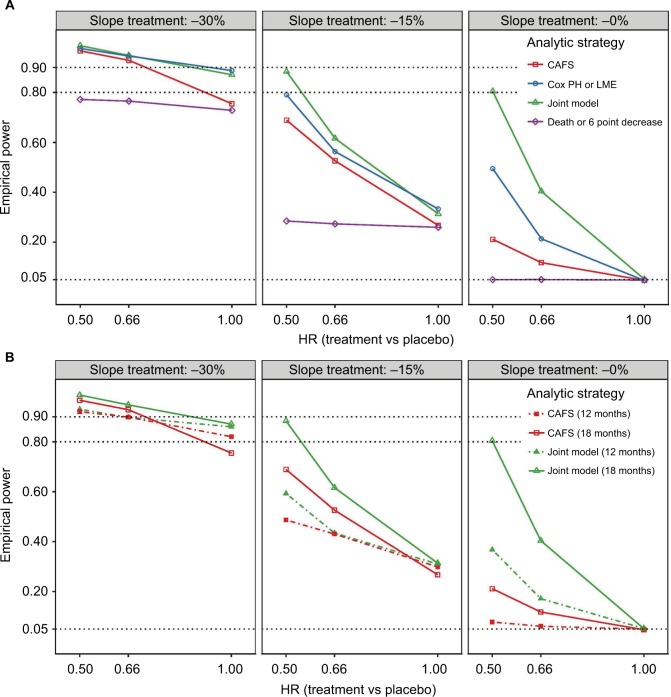
Empirical power of the four analytical strategies for different treatment scenarios. **Notes:** (**A**) Visual presentation of Table 3 of the empirical power after 18 months of follow-up. Panels from left to right show the effects of different treatments on functional decline (reduction in ALSFRS-R slope, *β*); on the *x*-axis are the treatment effects on survival (reduction in hazard rate, *γ*). (**B**) Direct comparison between the CAFS and the joint model for 18 months (solid lines) and 12 months (dashed lines) of follow-up. **Abbreviations:** ALSFRS-R, revised amyotrophic lateral sclerosis functional rating scale; CAFS, combined assessment of function and survival; HR, hazard ratio; LME, linear mixed effects; PH, proportional hazard.

Figure 3 visualizes the numerical results from Tables 3 and S2 for the four simultaneous analyses of survival and function. The joint model analysis is the most consistent strategy across all scenarios and superior in power compared to other methods. The composite end point time-to-6 point decrease or death fails to detect effects on survival; due to the large number of events generated by the ALSFRS-R (80%–90% had an event after 18 months), the relatively small increase in number of deaths minimally impacts the model and thus fails to affect empirical power. The composite end point also exhibits less power for detecting exclusive functional effects and increases false-negative rates in all treatment scenarios. The CAFS is underperforming in comparison with both the joint model and Cox or LME test, especially when there is only a treatment effect on survival (Figure 3A, right panel). In the scenarios where there is a simultaneous effect on survival and function (Figure 3A, left panel), differences between the three strategies are less extensive. Nevertheless, in a scenario with a 15% reduction in ALSFRS-R decline and 34% decline in hazard (HR = 0.66), the CAFS would require 576 patients for an 18-month trial, whereas the joint model would require 464 patients (a reduction of 19%) to detect the same treatment effect with 80% power.

### Paradoxical effect of follow-up time in CAFS

Interestingly, in the scenarios where there is an exclusive functional benefit, the CAFS is the only strategy that exhibits more power at 12 months than at 18 months (Figure 3B, left and middle panels). As the CAFS distinguishes between patients who die and survive, when a treatment exclusively affects functional decline, the surviving patients in the treatment arm are primarily driving the measured effect in the CAFS ranking system (Figures S3 and S4). As the proportion of patients alive is greater at 12 months, the number of higher ranking patients becomes larger, which in turn increases the contrast between treatment arms and subsequently the power. Vice versa, when there is an exclusive survival benefit, the deceased patients primarily affect the CAFS. As this is a proportionally smaller group than the surviving group, the treatment effect is diluted, which decreases the power of the CAFS to detect exclusive survival benefits. In the joint model and the omnibus Cox or LME test, this proportional imbalance is absent and inference is based on both outcomes.

## Discussion

In this simulation study, we have evaluated multiple strategies for the simultaneous analysis of function and mortality in confirmatory ALS clinical trials. The joint model analysis is the most consistent method across all treatment scenarios and can offer considerable improvements in statistical power or detect smaller treatment effects with identical sample sizes. In case of an exclusive survival benefit of treatment, incorporating the joint modeling framework could even enhance trial design over classical methods. Importantly, the joint model analysis performs similar to the CAFS in scenarios when there are large combined functional and survival benefits, but circumvents the shortcomings of the CAFS when there is an exclusive survival benefit.

In other fields, such as HIV or oncology, joint modeling has been extensively used to, for example, assess survival-adjusted CD4 counts or to model longitudinal tumor volume and tumor-related mortality.^10^,^14^,^19^,^20^ For ALS clinical trials specifically, joint models are relatively uncommon and have previously only been applied to adjust for informative censoring in longitudinal ALSFRS-R patterns.^1^,^15^ In the study by Healy and Schoenfeld,^1^ the joint model is compared with the CAFS for various treatment scenarios. However, they assessed the longitudinal arm of the joint model and ignored the survival component.^1^ Any direct comparison between the joint model and the CAFS to detect combined treatment effects is, therefore, lopsided. In our study, we balanced the comparison between the CAFS and the joint model by developing an omnibus test that summarizes the full treatment effect in a joint modeling framework. Not only is the joint model analysis the most powerful approach, it also removes the proportional imbalance between the survival and function end points seen in the CAFS analysis (Figures S3 and S4).

For trials designed on an exclusive mortality benefit of treatment, the joint model analysis may offer considerable improvements, even over classical analyses. For instance, redesigning the riluzole trial using the observed PRO-ACT survival probabilities,^13^ a Cox model requires ~1,282 patients, whereas the CAFS requires 3,922 patients and the joint model requires 800 patients, to detect an HR of 0.67 after 18 months with 80% power.^1^ For trials designed on an exclusive functional benefit of treatment, our results indicate that the LME is the most powerful approach (Table 3). However, as the ALSFRS-R is limited by its questions, it may not capture other survival-related processes that could be simultaneously influenced by treatment (e.g., plasma creatinine).^21^ Solely assuming an exclusive functional treatment effect could thus under- or overestimate the full effect of treatment. Moreover, a priori, it is unknown how treatment will affect both outcomes, and therefore, a joint modeling approach may offer the safest strategy. Literature on clinical trial design using joint models is sparse;^14^ however, direct sample size calculations can be conducted.^22^ A complicating factor is that the joint model analysis (and CAFS) requires two treatment expectations (on mortality and function). This may increase the risk of making erroneous a priori assumptions. Our estimate of *α* (HR 0.83) may help investigators to determine realistic expectations, for which empirical power and sample sizes could also be simulated with our data-generating mechanism.

There are several limitations of this study, and joint models in general, that should be considered. First, as the omnibus joint model test summarizes the functional and survival data into one test statistic, clinical interpretation of the treatment effect is not straightforward. However, in contrast to the CAFS, the joint model provides the individual effects on mortality and function directly, whereby the directional effect of treatment on each outcome could be assessed without the need for further testing.^1^,^10^,^14^ Second, the performance of the strategies may change when using a different data-generating mechanism (e.g., incorporating an average nonlinear rate of decline on ALSFRS-R).^23^ We addressed this issue by comparing our estimates of empirical power of the CAFS with the reported estimates by Healy and Schoenfield,^1^ who used a different data-generating mechanism. As our empirical power estimates differ by only 2%, it is unlikely that our results will change drastically under different data-generating mechanisms.

Our study does not evaluate all combinations of survival and function in ALS clinical trials. In the trials investigating acetyl-l-carnitine and recombinant interferon beta-1a,^24^,^25^ self-sufficiency (based on individual ALSFRS-R items) or death was used as the primary outcome. As this outcome requires simulation of the separate ALSFRS-R items, including their individual and mutual relationships with survival, this would significantly increase the simulations’ complexity and computational time. Nevertheless, our proposed joint modeling method could be extended and improved. First, as was shown previously, dividing the ALSFRS-R total score into subdomains could reduce between-patient variability.^21^ Incorporating these subdomains separately in the joint modeling framework may increase empirical power. Moreover, incorporating other secondary outcomes, such as muscle strength, respiratory measures and biomarkers, might provide additional information about the treatment effect and could further improve trial design. At last, the time-to-event end point could be extended to other events, such as the loss of self-sufficiency and respiratory failure. However, as was seen in the 6-point decrease or death analysis, increasing the number of events does not necessarily increase statistical power. This phenomenon was described earlier when combining tracheostomy or noninvasive ventilation (NIV) with death, which increased variability in the survival end point and inflated the sample size.^7^ Therefore, equalizing a particular disease state with death may negatively affect trial end points.

## Conclusion

Our results show that joint models may offer considerable improvements for ALS clinical trials and may circumvent the pitfalls encountered by other end points. Optimizing trial end points is essential, as selecting suboptimal outcomes may disguise important treatment clues and further delay the development of effective drugs against this debilitating disease.

## Figures and Tables

**Table 1:** Null and alternative hypothesis of each analytical strategy to evaluate the combined treatment effect on survival and functional loss

Analytical strategy	Null hypothesis (*H*_0_)		Alternative hypothesis (*H*_1_)	
CAFS	*H*_0_ : *P*(*X*_treatment _> *X*_placebo_) = 0.5	The probability that a patient on treatment has a better outcome *X *(either survival or functional status) than a patient on placebo is 0.5	*H*_1_ : *P*(*X*_treatment _> *X*_placebo_) ≠ 0.5	The probability that a patient on treatment has a better outcome *X *(either survival or functional status) than a patient on placebo is higher or less than 0.5
Death or 6-point loss	*H*_0_ : *γ*_combined_ = 0	There is no difference between treatment arms in the probability of an event (death or 6-point loss) at any time point during follow-up	*H*_1_ : *γ*_combined_ ≠ 0	There is a difference between treatment arms in the probability of an event (death or 6-point loss) at any time point during follow-up
Joint model	*H*_0_ : *γ*_adjusted_ = 0 ∩ *β*_adjusted_ = 0	There is no difference between treatment arms in the probability of an event (death) at any time point, adjusted for functional status, and there is no difference between treatment arms in functional decline, adjusted for survival	*H*_1_ : *γ*_adjusted_ ≠ 0 ∪ *β*_adjusted_ ≠ 0	There is a difference between treatment arms in the probability of an event (death) at any time point, adjusted for functional status, and/or there is a difference between treatment arms in functional decline, adjusted for survival
Cox or LME test	*H*_0_ : *γ*_crude_ = 0 ∩ *β*_crude_ = 0	There is no difference between treatment arms in the probability of an event (death) at any time point, and there is no difference between treatment arms in functional decline	*H*_1_ : *γ*_crude_ ≠ 0 ∪ *β*_crude_ ≠ 0	There is a difference between treatment arms in the probability of an event (death) at any time point, and/or there is a difference between treatment arms in functional decline

**Notes:** Taking the exponent of *γ* will provide the HR (treatment vs control). The mean difference between treatment arms in rates of decline in ALSFRS-R is given by *β*. Note that the joint model incorporates the relationship between survival and function and thus adjusts the treatment effect, whereas in the Cox or LME test, this adjustment does not take place.

**Abbreviations:** CAFS, combined assessment of function and survival; LME, linear mixed effects; HR, hazard ratio; ALSFRS-R, revised amyotrophic lateral sclerosis functional rating scale.

**Table 2:** Baseline characteristics of the PRO-ACT database’s placebo patients

Patient characteristics	PRO-ACT database (N = 1,469)
Age, years	57 (11)
Sex, female	544 (37%)
Onset, bulbar	311 (21%)
Symptom duration at enrollment	
Mean, months	17 (7)
Distribution, n (%)	
<12 months	414 (28%)
12–24 months	815 (56%)
>24 months	240 (16%)
Diagnostic delay	
Mean, months	10 (6)
Distribution, n (%)	
<6 months	435 (30%)
6–12 months	597 (41%)
>12 months	434 (29%)
FVC, % predicted	91 (14)
ALSFRS-R total score	38 (5)
ΔFRS at baseline, points per month	−0.68 (0.5)
BMI, kg/m^2^	26 (4)
Plasma creatinine, μmol/L	70 (15)

**Notes:** Data are in mean (SD) or n (%). ΔFRS, $\frac{\text{ALSFRS}-R_{\text{baseline}}-48}{\text{Disease duration}}$

**Abbreviations:** FVC, forced vital capacity; ALSFRS-R, revised amyotrophic lateral sclerosis functional rating scale; BMI, body mass index; PRO-ACT, Pooled Resource Open-Access ALS Clinical Trials.

**Table 3:** Empirical power of each strategy for trials with a maximum follow-up duration of 18 months

Survival (HR, treatment vs placebo)	ALSFRS-R (slope reduction)	Cox model	LME model	Cox or LME model (omnibus test)	Joint model (omnibus test)	CAFS	Death or 6 pt.
1	0	0.050	0.052	0.046	0.051	0.047	0.048
0.66	0	0.273	0.058	0.214	0.404	0.118	0.050
0.5	0	0.586	0.053	0.495	0.804	0.212	0.050
1	15%	0.137	0.405	0.333	0.314	0.268	0.260
0.66	15%	0.566	0.406	0.563	0.616	0.526	0.274
0.5	15%	0.837	0.406	0.791	0.884	0.689	0.285
1	30%	0.378	0.921	0.888	0.871	0.755	0.729
0.66	30%	0.828	0.934	0.946	0.948	0.928	0.765
0.5	30%	0.958	0.931	0.976	0.987	0.966	0.772

**Note:** All simulations are based on a monthly follow-up scheme (SD = 0.16) with a maximum of 18 months of follow-up and 150 patients per treatment arm; each scenario was repeated 10,000 times (Figure S2).

**Abbreviations:** HR, hazard ratio; ALSFRS-R, revised amyotrophic lateral sclerosis functional rating scale; Cox, Cox proportional hazard; LME, linear mixed effects; CAFS, combined assessment of function and survival; pt., points decrease on ALSFRS-R from baseline.
